# Unveiling the silver lining: examining the effects of biogenic silver nanoparticles on the growth dynamics of in vitro olive shoots

**DOI:** 10.1186/s12934-024-02346-9

**Published:** 2024-03-13

**Authors:** Mohamed S. Hasanin, Sayed A.M. Hassan, A. M. AbdAllatif, Osama M. Darwesh

**Affiliations:** 1 Cellulose & Paper Department, National Research Centre, 33 El Bohouth St, P.O. 12622, Dokki, Giza, Egypt; 2 Tissue Culture Technique Lab, Central Laboratories Network and Pomology Dept, National Research Centre, Dokki, Cairo, 12622 Egypt; 3 Pomology Dept, Faculty of Agriculture, Cairo University, Giza, Egypt; 4 Agricultural Microbiology Dept, National Research Centre, Dokki, Cairo, 12622 Egypt

**Keywords:** *Olea europaea* L., AgNPs, Micropropagation, n vitro, Nanotechnology

## Abstract

The current study aimed to evaluate the effects of biogenic silver nanoparticles (AgNPs) on growth behavior and leaf anatomy of in vitro growing shoots of ‘Picual’ and ‘Dolce’ olive cultivars. Biosynthesis of AgNPs was carried out using the cell-free filtrate of *Fusarium oxysporum*. The dimension and shape of the synthesized AgNPs have been analyzed using spectroscopy and topography analysis tools, confirming that the biosynthesis of AgNPs is a crystalline nanostructure with an average particle size of 37 nm. The shoots of the selected olive cultivars were cultured on Rugini olive medium-supplemented AgNPs at 0, 10, 20, and 30mg L^− 1^. The effect of genotypes on shoot multiplication was significant, ‘Picual’ recorded higher values of shoot growth parameters compared with ‘Dolce’ cultivar. Adding AgNPs to the culture medium significantly affected the growth of in vitro olive shoots. AgNPs at 20 and 30mg L^− 1^ produced higher values of the number of shoots, shoot length, and leaf number of Picual cv. compared with the control treatments, but the higher AgNPs concentration harmed the growth parameters of Dolce cv. and recorded lower growth values compared with the lower concentration (10mg L^− 1^). AgNPs had a significant effect on leaf morphology and their anatomical structure. The current results showed that the stimulatory effect of AgNPs on shoot growth of in vitro olive shoots is highly dependent on plant genotype and nanoparticle concentration.

## Introduction

Micropropagation has been reported as a powerful technique for the mass production of pathogen-free and true-to-type olive cultivars and is a valuable tool for genetic improvement and germplasm conservation [1, 2]. In vitro, the propagation of olive is affected by several factors including plant genotype, growth medium, cytokinin type, and concentration [3, 4]. Microbial contamination, oxidations of phenolic compounds, and limited proliferation rates are the major problems of olive micropropagation [5, 6]. Recently, nanotechnology as a novel field of science has received much attention, it is expected to revolutionize a broad range of agriculture fields, e.g. of enhancing plant growth, crop yields, and control of pests and diseases [7–12]. The unique properties of nanoparticles make them highly applicable in various fields of biology, chemistry, physics and environmental science [13–16]. Nanoparticles greatly influence different aspects of plant morphology, physiology, and biochemistry [17]. In plant tissue culture, several reports have proved the positive effects of NPs on callus induction, shoot multiplication, and plant growth [18]. Among different types of nanoparticles, AgNPs have a wide application in various fields due to their superior physical, chemical and biological characteristic compared with bulk material [19, 20]. The key properties of AgNPs are highly reliant upon the particle’s shapes and size [21]. Moreover, the effect of AgNPs is highly dependent on plant genotype, the lite amount of AgNPs can reduce shoot length in *Hordeum vulgare*, while 100mg L^− 1^ of AgNPs has no significant effect on *Cucumis sativus* and *Lactuca sativa* [22]. Moreover, AgNPs at 10–20 mg L^− 1^ stimulation *Eruca sativa* growth while higher concentrations (100 mg L^− 1^ of AgNPs) recorded lower growth values [23]. Generally, nanoparticles are synthesized through chemical, mechanical and biogenic methods [24]. Recently biogenic synthesis of AgNPs has been reported by many researchers [25, 26]. Biogenic synthesis of nanoparticles can be performed using bacteria, fungi, and plants, or through their metabolism products, which act as reducing and stabilizing agents [27]. Green or biogenic synthesis is relatively simple, economical and environmentally friendly [28]. Due to the increase in the production and consumption of synthetic nanoparticles (NPs), the potential release into the environment is estimated to increase dramatically [29]. Several studies have demonstrated the phytotoxicity of higher concentrations of nanoparticles on plant tissue [30]. The majority of nanoparticles can change the morpho-anatomical, physiological, biochemical and genetic constitutions of the treated plant tissue [31]. NP treatments affect the mitotic activity and alter the protein and DNA profile in plants [32]. AgNPs phytotoxicity may be caused by the production of reactive oxygen species (ROS), which results in lipid peroxidation, proteins and DNA damage [33]. AgNPs induce variation in callus morphology, and anatomy and increased somaclonal variation in calli and regenerated shoots [34, 35]. Thus, the current study aimed to investigate the effect of biogenic AgNPs at different concentrations on the growth of in vitro ‘Picual’ and ‘Dolce’ olive shoots.

## Materials and methods

### Preparation of silver nanoparticles

All chemicals used in this study were of reagent grade and all solutions were prepared with deionized water; the biogenic AgNPs were synthesized using the cell-free filtrate of *F. oxysporum* [24]. Ten ml of the cell-free filtrate was added to 10 ml of 1 M silver nitrate (Merck-Darmstadt, Germany) solution in bottles with caps. The bottles were incubated under static conditions at room temperature for 24 h.

### Characterization of the synthesized nanoparticles

The prepared AgNPs were characterized using spectroscopy techniques including UV**-**visible spectroscopy; UV-visible spectra were measured using UV–Vis spectrophotometer (Jasco, V-630, Tokyo, Japan) in the range of 200-1000 nm. FT-IR spectra were measured using Impact-400 FT-IR spectrometer (Nicolet Analytical Instruments, 5225-1, Madison, USA) in the range of 400–4000 cm^− 1^. The topographical as well as the particle size determination were carried out using Field emission SEM coupled with energy dispersive X-ray analysis; Model Quanta 250 FEG (Field Emission Gun) attached with EDX Unit (Energy Dispersive X-ray Analyses) for EDX. Particle size and morphology of AgNPs have been analyzed using transmission electron microscopy (JEOL, JEM 2010, Japan).

### Plant materials and explants preparation

Active spring shoots were collected from mature olive trees of ‘Picual’ and ‘Dolce’, cultivars (grown at an olive collection farm, Faculty of Agriculture, Cairo University, Giza, Egypt). The defoliated olive shoots were divided into nodal segments and surface sterilized by 20% commercial bleach c (5.5% of NaOCl) for 10 min followed by mercuric chlorate (HgClO_2_), at 1000 mg L^− 1^ then rinsed with sterile water for 5 min. The collection of plant material complies with the guidelines of the Ethics Committee in the National Research Centre.

### Examine the effect of silver nanoparticles on the in vitro growth of olive shoots

Nodal segments of the selected cultivars were cultured on Rugini olive medium [36], supplemented with 2.5 mg L^-1^ zeatin, 30 g L^-1^ mannitol and 6 g agar L^-1^. Media pH was adjusted to 5.8 before adding agar and the media was autoclaved at 121 °C for 15 min. All cultures were maintained in a growth chamber at 25 ± 2 °C and 16 h photoperiod (provided with 40–60µmol m^-2^ s^-1^ cool-white fluorescent lamps). After four weeks the sprouted buds were transferred to fresh Rugini media supplemented with AgNPs at concentrations of 0, 10, 20 and 30 mg L^-1^, all media were supplemented with 30 g L^-1^ mannitol and 6 g agar L^-1^ and autoclaved as described above. Each treatment consisted of 20 jars, four single nodes cutting off the 2nd subculture was cultured on 200 ml glass jar containing 50 ml of semi-solid medium and maintained in the growth chamber at 25 ± 2 °C and 16 h photoperiod with 40–60µmol m^-2^ s^-1^ provided by cool-white fluorescent lamps, sub-culture was performed every four weeks. The number of shoots per explant, shoot length and number of leaves per shoot were recorded after the two sub-cultures.

### Examine the effect of silver nanoparticles on leaf morphology

Olive leaves were collected from 4 weeks of shoots; fully expanded leaves from healthy and uniform shoots were sampled; leaves photographs were captured by a digital camera (Panasonic WV-CP 220, Japan), and the obtained images were subjected to analysis by image analysis software (Digimizer version, 4.2.6.0, MedCalc Software Ltd) to calculate leaf length, width and area. To calculate leaf thickness; leaves were fixed in F.A.A (10 ml formalin, 5 ml glacial acetic acid, and 85 ml ethyl alcohol 70% solution for 48 h), and dehydrated through a series of ethyl and butyl alcohol and embedded in paraffin wax (melting point 56^o^C). Leaf sections (20 μm) were double stained in combination with crystal violet-erythrosine, cleared in xylene and mounted in Canada balsam. The leaf sections were examined under a light microscope (LEICADM750, Germany). Photographs were captured at 100X magnification with a digital camera (LEICA ACC50 HD, Germany). The obtained images were subjected to analysis by image analysis software (Digimizer version, 4.2.6.0, MedCalc Software Ltd) to calculate section thickness.

### Experimental design and statistical analysis

The experiment was carried out in a completely randomized design, the assumptions of normality were tested using Shapiro-Wilk’s test. Normally distributed data was subjected to a two-way analysis of variance to investigate the effect of olive genotype, nanoparticle treatments and their interaction. Analysis of variance was performed using MSTAT-C statistical package software. The mean and standard error (± SE) were calculated from three replicates per treatment and the significant differences within and between treatments were assessed using the LSD test at a significance level of 0.01.

## Results and discussion

### Spectroscopy characterizations

**Fig. 1:**
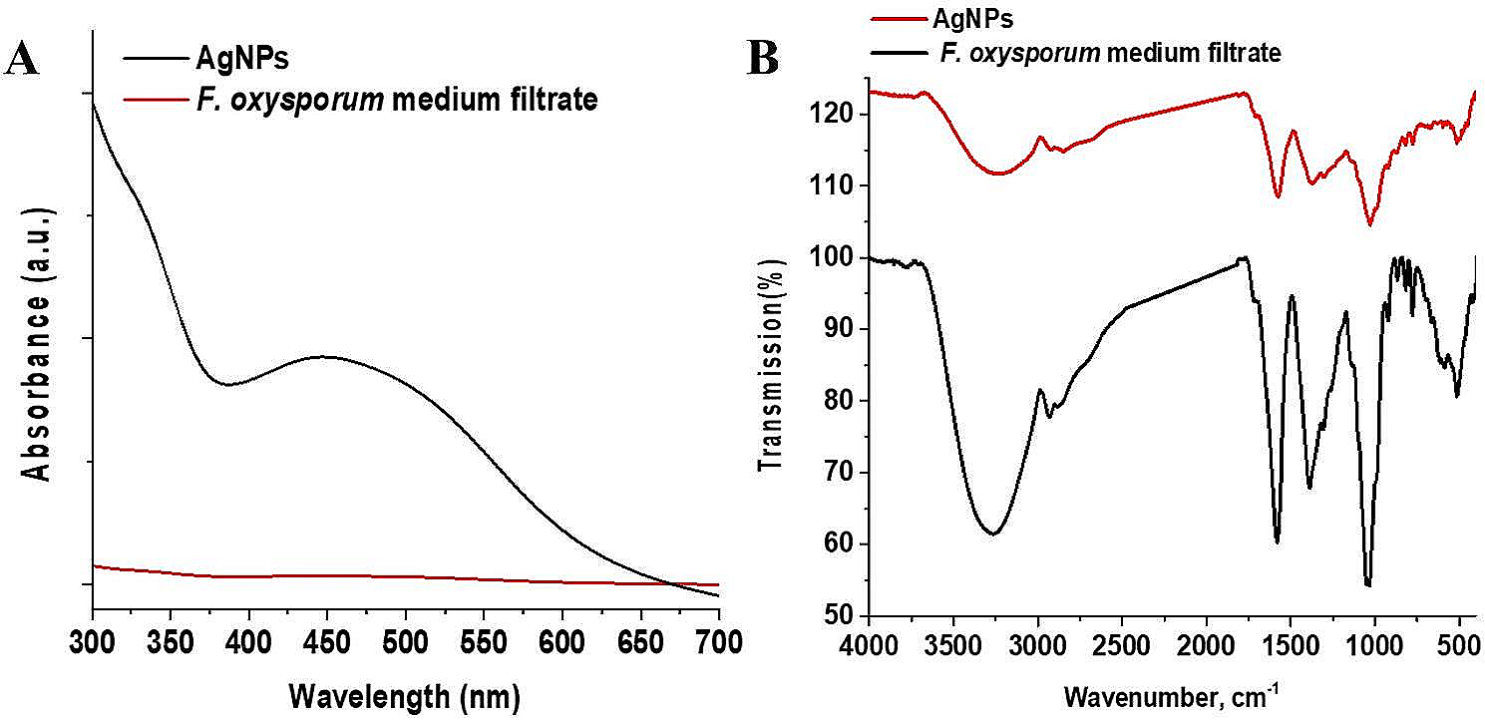
UV–vis spectra (**A**) and FTIR spectra (**B**) of *F. oxysporum* medium filtrate and biosynthesis AgNPs

The characterizations of the biosynthesis AgNPs were carried out *via* spectroscopy in comparison with the cell-free filtrate of *F. oxysporum*. The UV-vis spectroscopy spectra are shown in Fig. 1A. The cell-free filtrate of *F. oxysporum* did not show any specific bands’ overall spectra range. On the other hand, the spectrum of biosynthesis AgNPs showed a broad surface plasmon resonance (SPR) peak for AgNPs at around 450 nm which was affirmed by the color change from colorless to reddish-brown due to the excitation of surface plasmon resonance (SPR) phenomena of AgNPs [37].

Another informative spectroscopy tool used in the characterization of biosynthesized AgNPs and *F. oxysporum* medium filtrate function groups is FTIR (shown in Fig. 1b). The spectra were illustrated in Fig. 1A where the *F. oxysporum* medium filtrate spectrum was illustrated a characteristic bands at 3276, 2931, 1580 and 1049 cm^− 1^ which corresponds to hydroxyl groups vibration stretching, C-H groups asymmetric stretching vibration of aliphatic groups, polyphenol skeleton of aromatic structures, C-O carbohydrate bond, respectively [38, 39]. In the country, FTIR analysis is used to elucidate the functional groups involved in the reduction/capping of the silver ions to the nano-scale. The AgNPs spectrum illustrated significant changes during AgNPs biosynthesis. The hydroxyl group band was shifted to the lower frequency as the small broadband. The C-H group asymmetric stretching vibration was shifted to a lower frequency as a small band. Additionally, the band of polyphenols appeared as a small sharp band as well and the carbohydrate linkage band was shifted to lower frequency. Moreover, two new bands were observed at the fingerprint region for out-of-plane ring bending at 509 and 449 cm^− 1^ [40]. These above observations affirmed the involvement of the *F. oxysporum* medium filtrate in the biosynthesis of the AgNPs *via* its function groups.

### Topographical surface and particle morphology

**Fig. 2:**
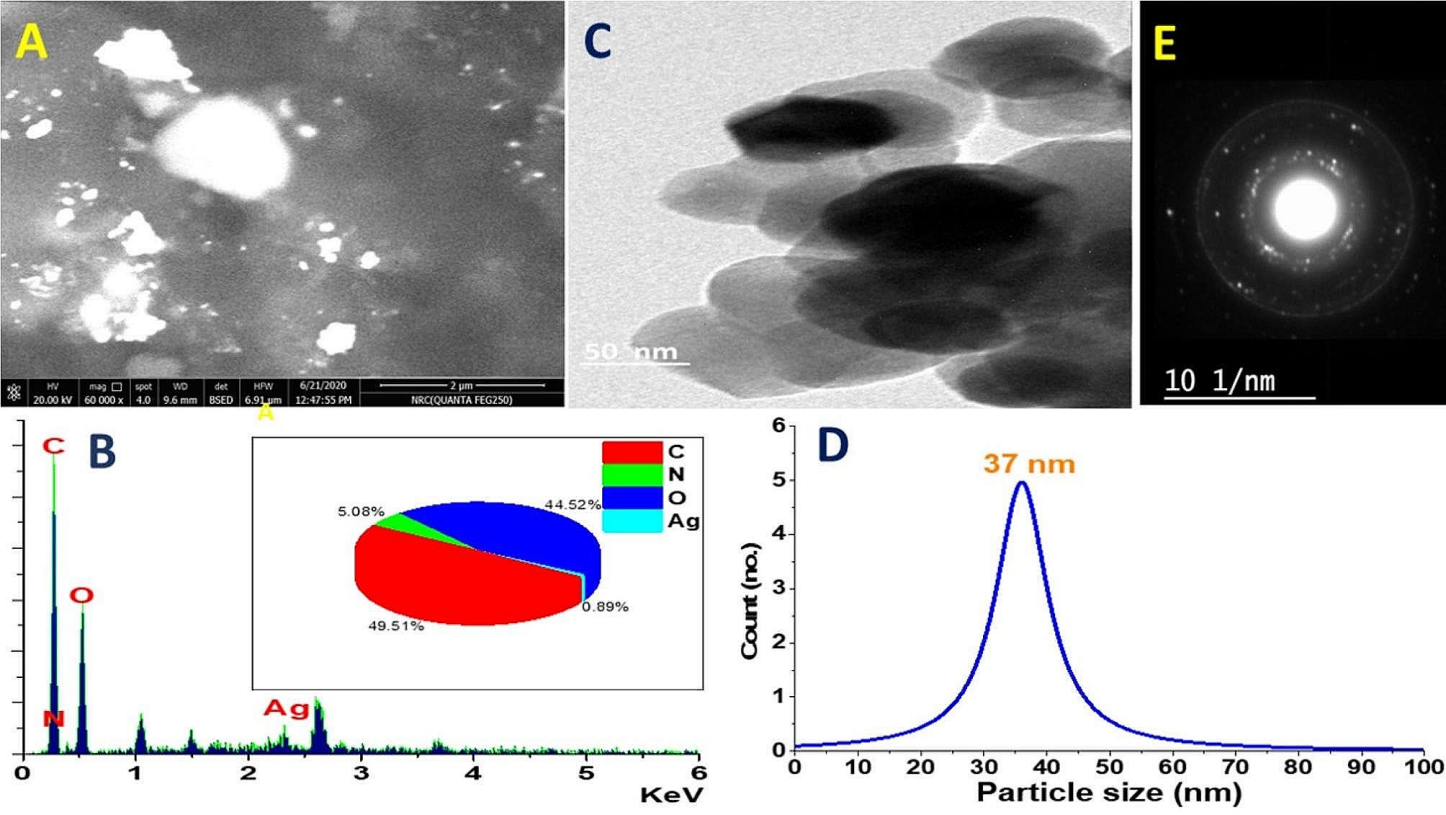
: Topography study of biosynthesis AgNPs: (**A**) SEM image, (**B**) EDX chart, (**C**) TEM image, (**D**) particle size distribution histogram, and (**E**) SAED pattern

The topography of the materials emphasizes the crystalline structure as well as illustrates the behavior of particles to each other as shown in Fig. 2. The SEM image and EDX chart are illustrated in Fig. 2A and B, respectively. The SEM image was clear a metallic sheen spots which may be related to the aggressions of the AgNPs. Moreover, The EDX chart informed the presence of Ag ions and some other ions including carbon, nitrogen, and oxygen which referred to the *F. oxysporum* medium main components. The selected-area electron diffraction pattern (SAED) confirmed the polycrystalline structure of the biosynthesis AgNPs with high crystallinity as shown in the intensity of the ring spots (Fig. 2E). In addition, the TEM images of the synthesized AgNPs were shown in Fig. 2C and confirmed that the average particle size is 37 nm; AgNPs were spherical and well scattered in the solution. These results are in agreement with the spectroscopy analysis and all affirmed the nanostructure of the AgNPs. The mean size of the obtained nanoparticles is comparable to the particle size that has been reported in previous studies for silver nanoparticles [41].

### Effect of silver nanoparticles on in vitro growth of olive shoots

**Fig. 3:**
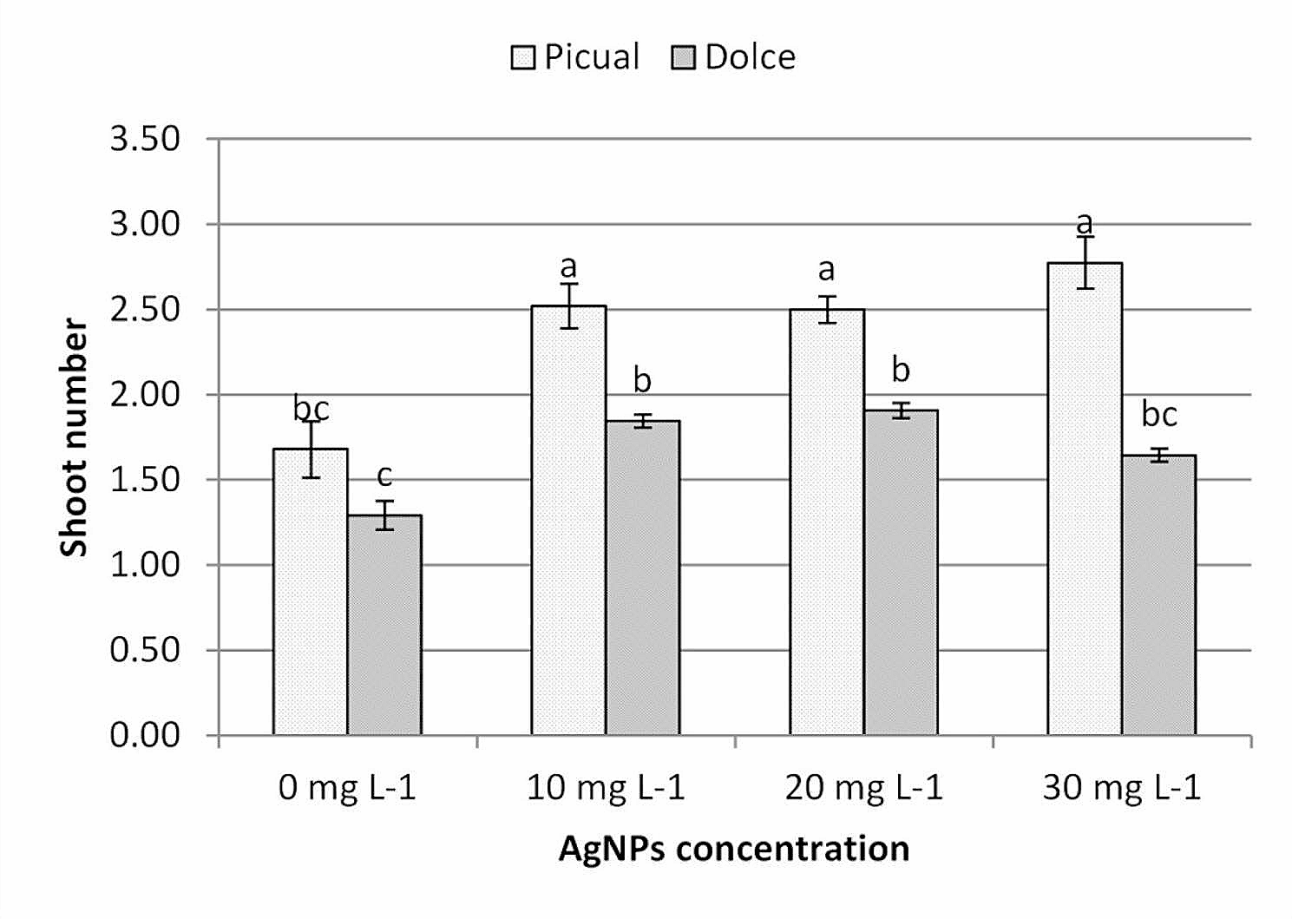
The effect of AgNPs concentration on shoot number of different olive cultivars; means followed by different letters are significantly different at *P* ≤ 0.01

According to the data illustrated in Fig. 3 in vitro growth of olive shoots was significantly affected by both of plant genotype and AgNPs concentration in the growth medium. Picual cv. recorded a higher number of shoots per explant compared with Dolce cv., the highest number of shoots was recorded for Picual shoot growing in medium supplemented with AgNPs at 30mg L^− 1^ (2.77 ± 0.15) followed by both 10 and 20 mg l^− 1^ (2.52 ± 0.13 and 2.50 ± 0.08 respectively); control treatments recorded statistically the lowest value of number of shoots.

**Fig. 4:**
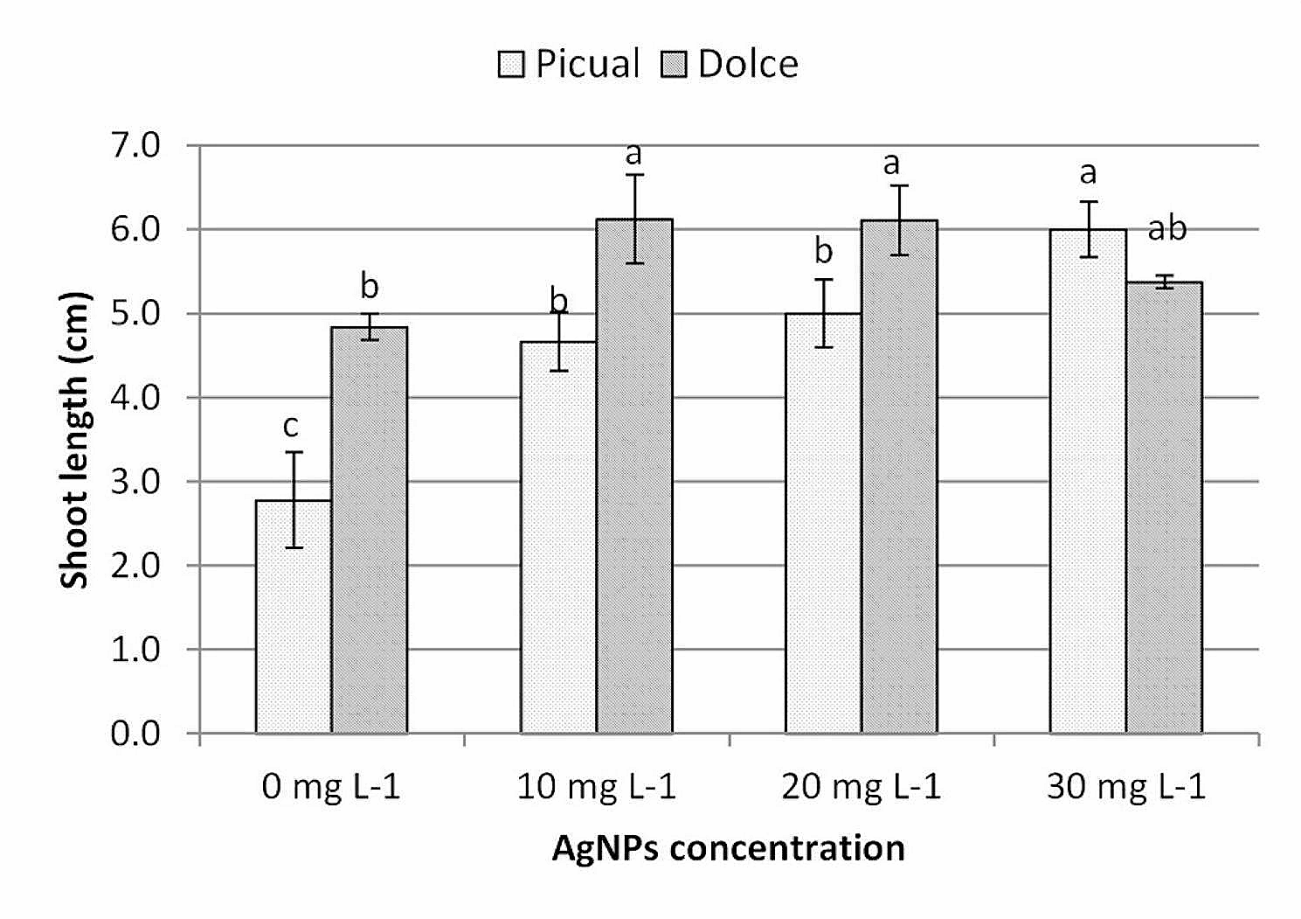
The effect of AgNPs concentration on shoot length of different olive cultivars; means followed by different letters are significantly different at *P* ≤ 0.01

As shown in Fig. 4 Dolce cv. recorded higher shoot length compared with Picual cv., it is evident that the addition of nanoparticles to the culture medium significantly enhanced shoot growth of in vitro cultured olive shoots compared with the control treatment; the average shoot length of Picual cv. was increased from 2.78 cm in control treatment to 6.00 cm in AgNPs treatments at 30mg L^− 1^; using AgNPs caused an increase in shoot length of Dolce cv., from 4.84 cm in the control treatment to 6.13 cm in AgNPs treatments at 10mg L^− 1^, further increase of AgNPs slightly reduced Dolce shoot length. The differences between 10, 20 and 30 mg AgNP treatments were non-significant in both cultivars.

**Fig. 5:**
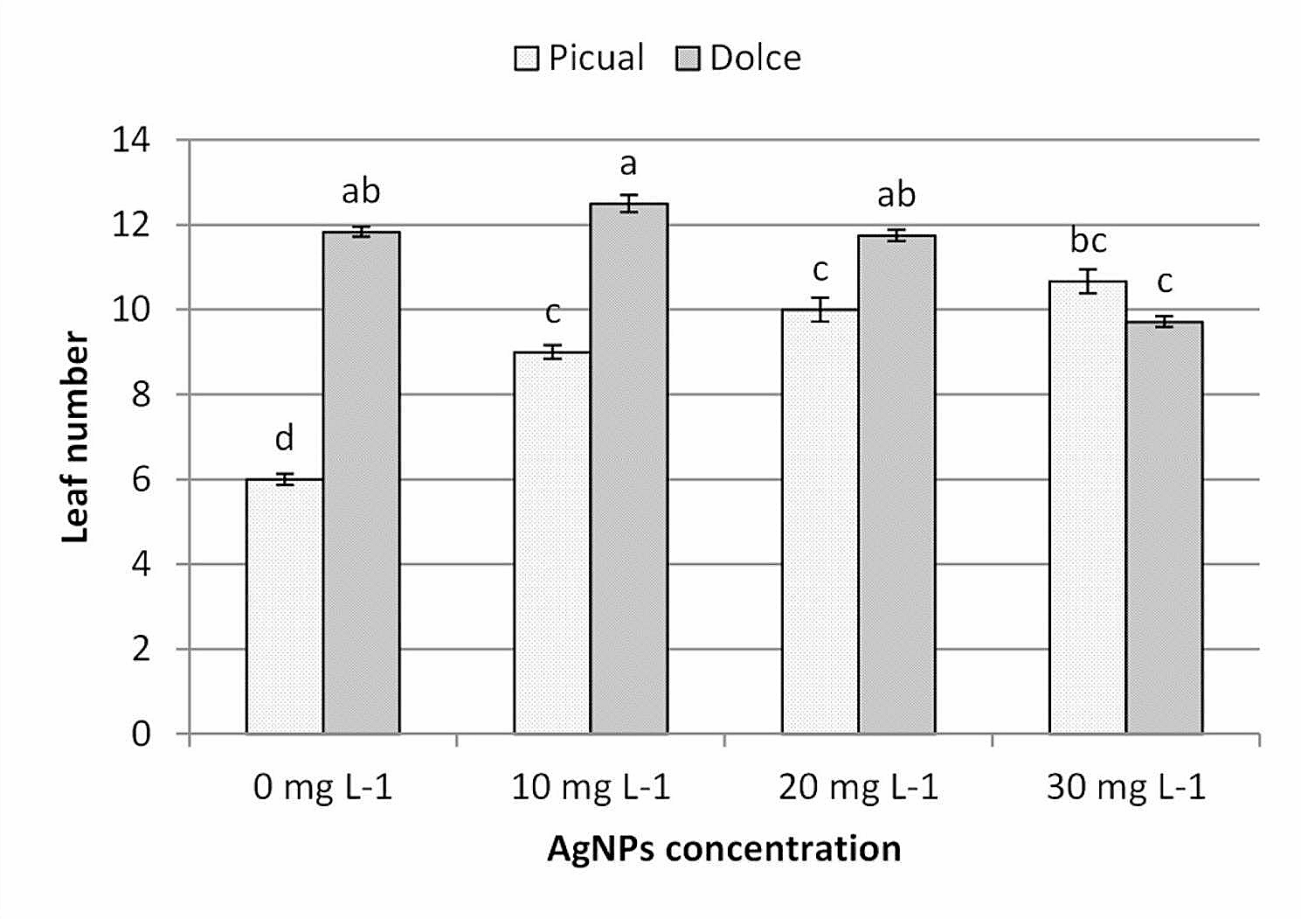
The effect of AgNPs concentration on leaves several different olive cultivars; means followed by different letters are significantly different at *P* ≤ 0.01

According to the data presented in Fig. 5 Dolce cv., recorded a higher value of leaf number compared with Picual cv., leaf number of Picual cv. showed a significant incremental trend with increasing of AgNPs concentration in culture media up to 30mg L^− 1^. AgNPs at 10 mg L^− 1^ recorded the highest leaf number Dolce cv.; while the higher concentration (20 and 30mg l^− 1^) recorded a lower leaf number per shoot of Dolce cv. Our results confirm that AgNPs can lead to an improvement in plant growth, which is consistent with our previously published data [42, 43]. The obtained results indicated that AgNPs had a stimulatory effect on shoot regeneration of in vitro growing olive shoots of both cultivars and the impact of nanoparticles on shoot growth was highly dependent on NP concentrations [44, 45]. Silver ion has a positive effect on in vitro growing shoots, e.g. increased survival and delayed explants senescence [46], improved organogenesis, increased shoot multiplication rate and improved shoot growth [47]. Shoot growth and number of shoots per explant were increased in *Brassica juncea, Tecomella undulate* Roxb., and *Vanilla planifolia* cultured on medium supplemented with AgNPs [18], which was attributed to the effect of Ag^+^ as an ethylene blockage agent; as the addition of cytokinin to growth media is known to stimulate ethylene production; silver ions would result in blocking of ethylene action and promote shoot regeneration and delay explant senescence. Moreover, Syu et al. (2014) [48] indicated that AgNPs inhibited ethylene action through reduced the expression of *ACC* synthase *7* and *ACC* oxidase *2*.

### Effect of silver nanoparticles on leaf morphology

**Fig. 6:**
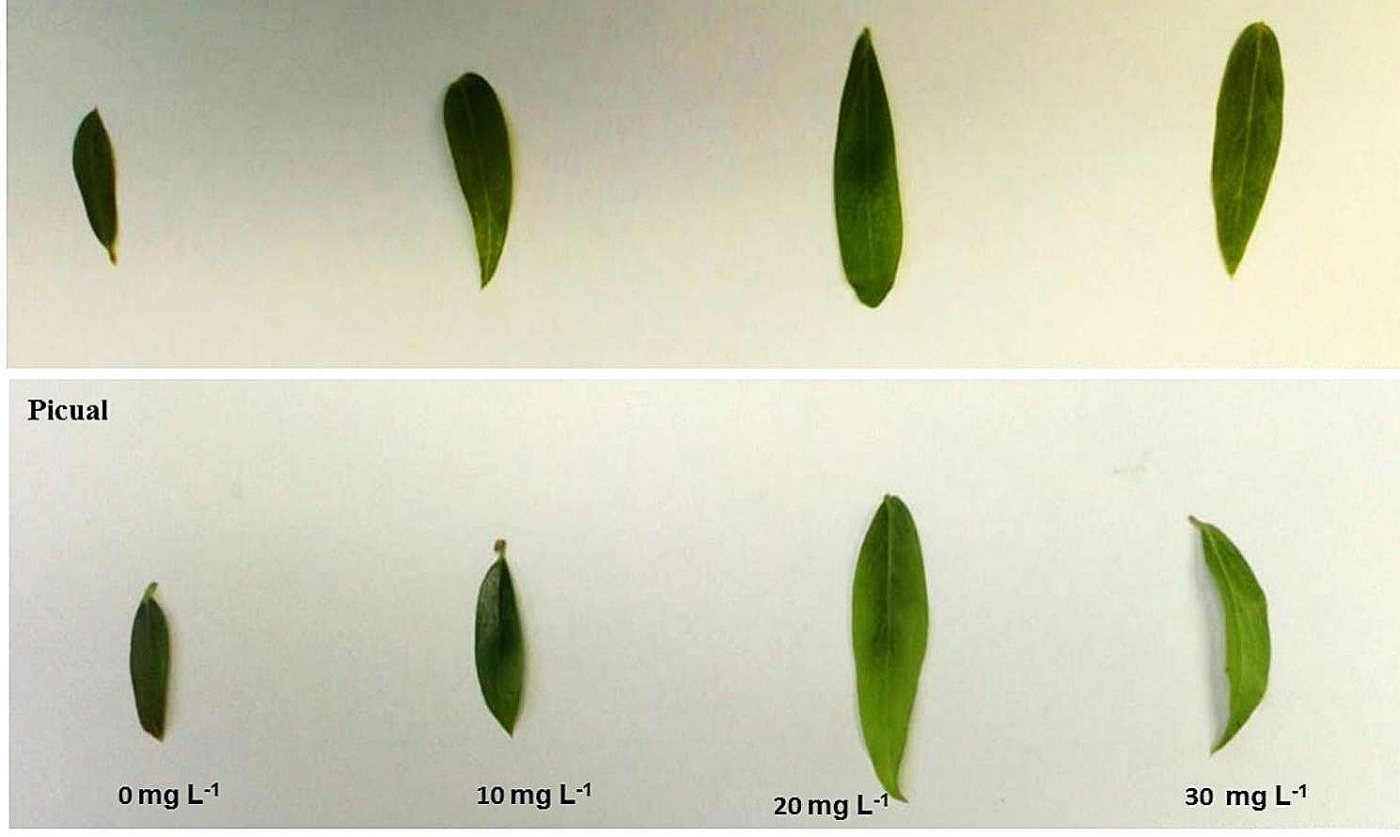
The effect of AgNPs concentration on leaves morphology of different olive cultivars

**Fig. 7:**
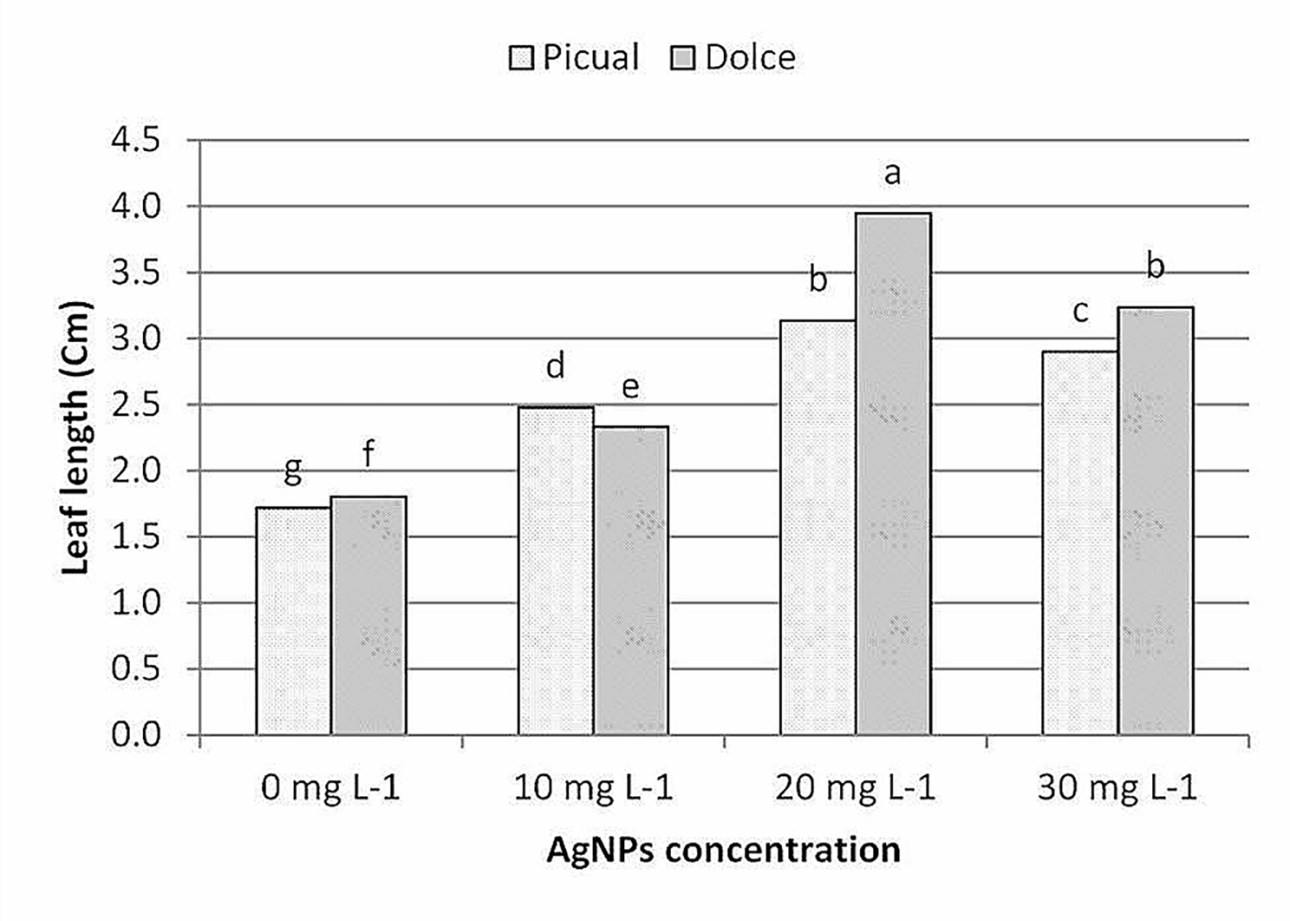
The effect of AgNPs concentration on leaf length of different olive cultivars; means followed by different letters are significantly different at *p* ≤ 0.01

**Fig. 8:**
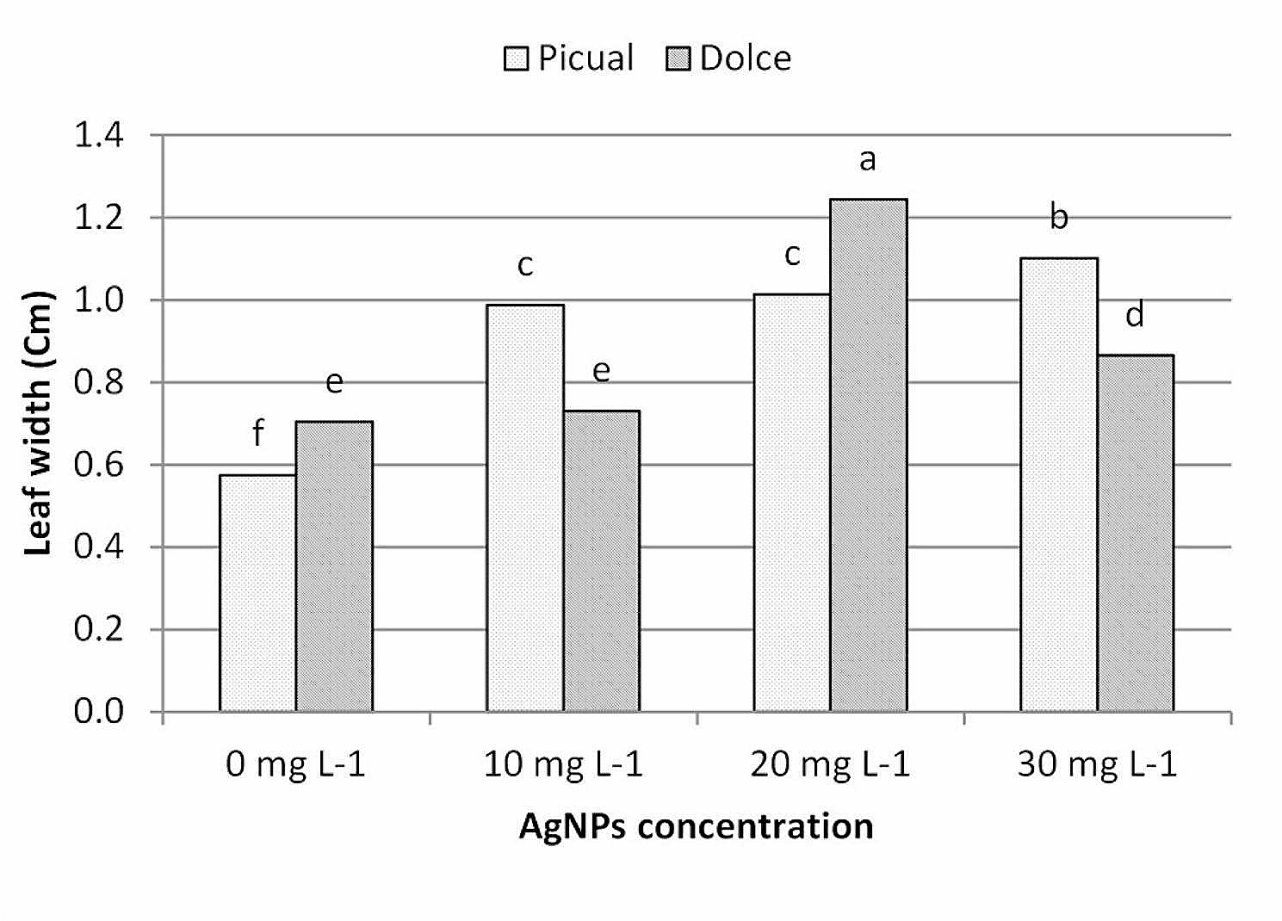
The effect of AgNPs concentration on leaf width of different olive cultivars; means followed by different letters are significantly different at *p* ≤ 0.01

**Fig. 9:**
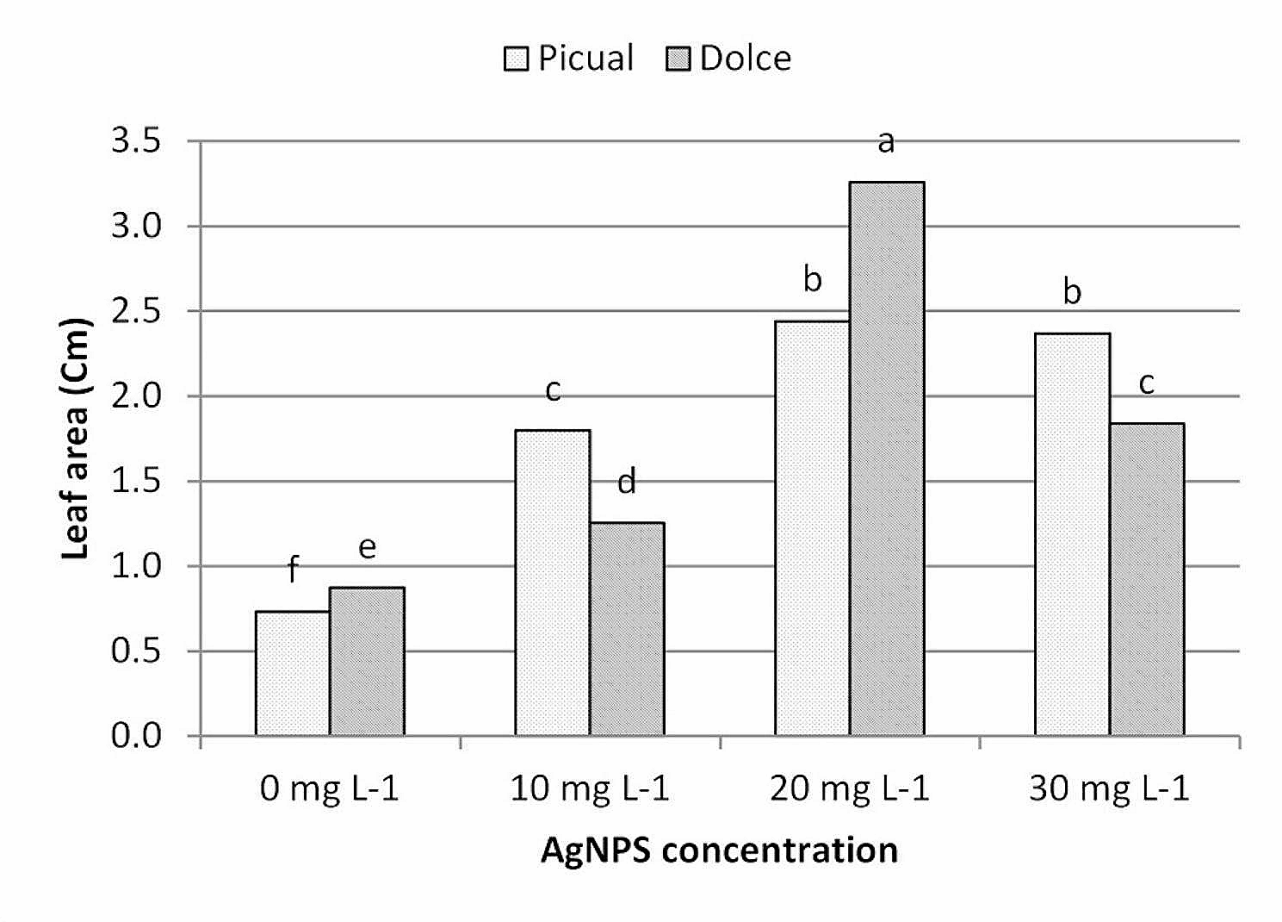
The effect of AgNPs concentration on leaf area of different olive cultivars; means followed by different letters are significantly different at *p* ≤ 0.01

The results of leaf morphological parameters showed that AgNPs exhibit growth-stimulating activity, analysis of morphological data showed that AgNPs treatments had a significant effect on leaf morphological traits; as shown in Figs. (6, 7, 8, 9); leaf length, width and area were significantly greater for the AgNPs treatments compared with the control treatments. The differences in leaf measurements were more obvious in Dolce cv. compared with Picual cv., the highest value of morphological leaf parameters was recorded for AgNPs at 20mg L^− 1^, increasing the concentration of AgNPs up to 30mg L^− 1^ decreased leaf parameters while the lowest was recorded for the control samples.

**Fig. 10:**
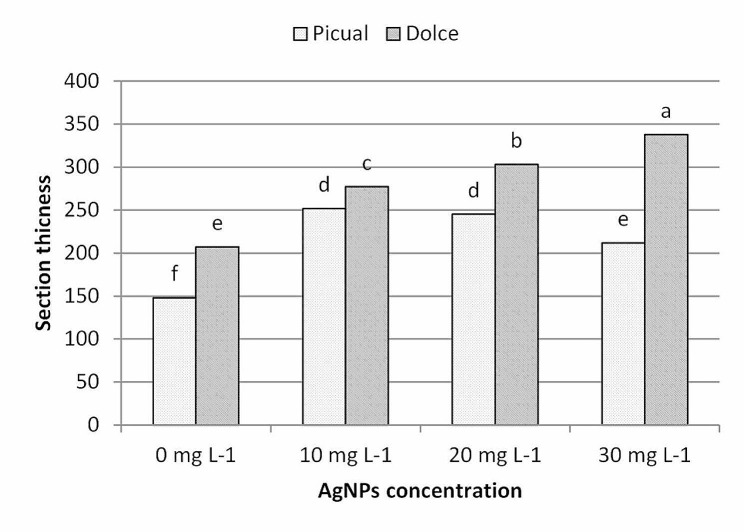
The effect of Ag NPs concentration on leaf section thickness of different olive cultivars, means followed by different letters are significantly different at *p* ≤ 0.01

As shown in Fig. 10 addition of nanoparticles to the culture medium significantly increased the leaf section thickness of in vitro cultured olive shoots compared with the control treatment; the average section thickness of Picual cv. was increased from 147.84 μm in the control treatment to 251.78 μm in 10mg L^− 1^ AgNPs treatments, than further increase of AgNPs slightly reduced leaf section thickness and recorded 211.746 μm at 30mg l^− 1^; also, using AgNPs showed an upward increase trend in section thickness of Dolce cv., section thickness increased from 207.18 μm in the control treatment to 337.84 μm in AgNPs treatments at 30mg L^− 1^. Analysis of morphological parameters showed that AgNPs exhibit growth-stimulating activity and increasing concentration of AgNPs improved leaf morphological measurements. According to our results, AgNPs treatments had almost a stimulatory effect on the growth parameters of in vitro olive shoot, which varied according to the genotype; similar results were reported for different plant species including *Brassica juncea* [49], *Beta vulgaris* [50], *Phaseolus vulgaris* [51]. The impact of AgNPs on higher plants appears to depend on the species, age of plants, particle size and concentration. The application of nanoparticles should be optimized to improve plant growth and avoid the toxic effect of higher doses; the higher concentrations of NPs had adverse effects on cell viability, shoot growth and plant regeneration [52]. Toxicity caused by NPs in plants leads to morphological changes and abnormalities in cell growth [53]. Plant growth, biomass production, and leaf area are commonly affected by AgNPs treatment [54]. Higher concentrations of AgNPs significantly decreased plant biomass, inhibited shoot growth, and reduced root elongation [55]. Our results showed that AgNPs at the used concentrations had low toxicity on olive shoots; this may be attributed to the lower toxicity potential of the biogenic AgNPs. The toxicity of AgNPs depends on particle size, concentration and the nature of coating materials [56]. Salama (2012) [57] reported that low concentrations (20 and 40 ppm) of AgNPs have stimulatory effects on the growth of *P*. *vulgaris* and maize, whereas higher concentrations (100 ppm) had inhibitory effects. Moreover, biogenic AgNPs had a lower toxicity potential compared with the bulk silver ions and chemically synthesized AgNPs [58].

## Conclusion

The biosynthesis of AgNPs is a promising ecofriendly and economical process that can be carried out using low equipment requirements with excellent crystalline nanostructure. According to the obtained results, AgNPs showed a significant effect on the growth of in vitro growing olive shoots, which was highly, affected by plant genotype and AgNP concentrations.

## Data Availability

No datasets were generated or analysed during the current study.
